# Nano-Cilostazol Mitigates Cisplatin-Induced Nephrotoxicity in Rats via Modulation of Oxidative Stress, Apoptosis, Pyroptosis, and miRNA-155 Signaling

**DOI:** 10.3390/antiox15030315

**Published:** 2026-03-02

**Authors:** Hebatallah M. Saad, Enas I. El Zahaby, Alyaa R. Salama, Ahmed M. Elgazzar, Hisham A. Nematalla, Mona Elharoun, Nihal E. Amer, Aml E. Hashem, Omnya Elhussieny, Ahmed Elsawasany, Salman A. A. Mohammed

**Affiliations:** 1 Department of Pathology, Faculty of Veterinary Medicine, Matrouh University, Marsa Matruh 51511, Egypt; heba.magdy@mau.edu.eg; 2 Department of Pharmaceutics, Faculty of Pharmacy, Delta University for Science and Technology, Gamasa 35712, Egypt; enas.elzahabi@deltauniv.edu.eg; 3 Department of Pathology, Faculty of Veterinary Medicine, Alexandria University, Alexandria 21944, Egypt; alyaa.salama@alexu.edu.eg; 4 Department of Veterinary Forensic Medicine and Toxicology, Faculty of Veterinary Medicine, Alexandria University, Alexandria 21944, Egypt; elgazzar@alexu.edu.eg (A.M.E.); a_elsawasany@alexu.edu.eg (A.E.); 5 Department of Pharmacology and Toxicology, Faculty of Pharmacy, Damanhour University, Damanhour 22514, Egypt; hisham.nematalla@pharm.dmu.edu.eg (H.A.N.); mona.elharoun@pharm.dmu.edu.eg (M.E.); 6 Faculty of Pharmacy and the Research & Innovation Hub, Alamein International University, Alamein 51718, Egypt; 7 Benha University Hospital, Benha University, Benha 13518, Egypt; nihalamer2301@gmail.com; 8 Departement of Biochemistry, Faculty of Veterinary Medicine, Alexandria University, Alexandria 21944, Egypt; amlhashem@alexu.edu.eg; 9 Department of Histology and Cytology, Faculty of Veterinary Medicine, Matrouh University, Marsa Matruh 51511, Egypt; omnya.elhussieny@mau.edu.eg; 10 Department of Pharmacology and Toxicology, College of Pharmacy, Qassim University, Buraydah 51452, Saudi Arabia

**Keywords:** cisplatin, nephrotoxicity, oxidative stress, spanlastic cilostazol

## Abstract

Background: This study investigated the renoprotective potential of Nano-Cilostazol against cisplatin (CIS)-induced renal injury in male rats and explored its molecular mechanisms. Our results showed that Nano-Cilostazol has a favorable physicochemical characteristic, including a mean particle size of approximately 101 nm, narrow polydispersity, and high stability. FTIR analysis indicated successful drug entrapment, preserving functional groups and enhancing hydrogen bonding. Docking analysis showed that cilostazol had stronger binding affinities than disulfiram against seven acute kidney injury-related targets. Interaction profiling confirmed stable binding through hydrogen bonding, hydrophobic, and π-interactions with BAX, ASC, GSDMD, KIM-1, JAK2, NLRP3, and miRNA-155. In vivo, CIS administration led to marked renal dysfunction, showing up as significant elevations in serum urea, creatinine, cystatin-C, CRP, and NGAL which indicated by severe histopathological damage. Co-treatment with Nano-Cilostazol significantly lessened renal functional impairment biochemically and histopatologically. Nano-Cilostazol markedly reduced lipid peroxidation and oxidized glutathione while also restoring antioxidant defenses like superoxide dismutase and catalase, with total and reduced glutathione. Additionally, Nano-Cilostazol attenuated renal inflammation, inhibiting NF-κB activation, lowering pro-inflammatory cytokines (TNF-α and IL-1β), and downregulating inflammatory and injury-related genes. CIS-triggered apoptotic signaling was also mitigated, shown by increased caspase-3 and BAX expression with downregulation of BCL-2. Nano-Cilostazol significantly inhibited apoptosis and pyroptosis (NLRP3, ASC, GSDMD)-related pathways, modulated JAK2/STAT3 signaling, and downregulated miRNA-155 expression. In conclusion, Nano-Cilostazol offers potent protection against cisplatin-induced nephrotoxicity.

## 1. Introduction

Cisplatin (CIS) remains one of the most effective and widely used chemotherapeutic agents for treating solid cancers [1]. A study by Xiao et al. [2] has significant translational implications for optimizing lung cancer treatment strategies. Specifically, the data suggest that the combination of celecoxib and cisplatin can synergistically enhance antitumor efficacy in tumors harboring wild-type p53 by promoting apoptosis through upregulation of PUMA and facilitating p53 nuclear translocation. Despite its potent anti-tumor activity, CIS use is often limited by its serious side effects, foremost among them being nephrotoxicity [3]. CIS-induced renal injury is characterized by a drop in renal function attributable to damage to renal tubular epithelial cells, particularly in the proximal tubules, resulting in impaired filtration, accumulation of waste products, and ultimately renal failure [4]. Pathogenesis comprises numerous mechanisms, including oxidative stress, inflammation, vascular injury, and apoptosis, which collectively contribute to cellular damage and loss of renal function [5].

Clinically, this damage is typically evaluated by blood urea nitrogen (BUN) and serum creatinine; however, these markers have limitations in sensitivity and timeliness, often reflecting injury only after significant renal damage has occurred [6]. Nevertheless, cystatin-C and neutrophil gelatinase-associated lipocalin (NGAL) have emerged as reliable and early biomarkers of acute kidney injury (AKI) [7]. Elevated serum cystatin-C levels have been revealed to correlate strongly with renal tubular damage and serve as an early marker for AKI onset [8]. Similarly, NGAL, a glycoprotein produced predominantly by neutrophils and epithelial cells, is rapidly released into the bloodstream and urine following renal tubular injury, making it a highly sensitive early biomarker of nephrotoxicity [9].

Pyroptosis is a highly inflammatory form of cell death that is described by cell swelling, lysis, and the release of inflammatory molecules [10]. NOD-like pyrin domain-containing protein 3 (NLRP3) inflammasome, Gasdermin D (GSDMD), and miRNA-155 are cardinal players in pyroptosis [11]. Targeting the inflammasome and miR-155 or their downstream pathways may offer a potential therapeutic strategy for AKI.

Cilostazol, a phosphodiesterase III inhibitor with vasodilatory, antiplatelet, antioxidative, and anti-inflammatory effects, is a promising candidate as a renoprotective agent [12,13]. Cilostazol is classified as a class II drug according to the biopharmaceutical classification systems (BCSs). The combination of low aqueous solubility and high permeability presents substantial formulation challenges, resulting in suboptimal dissolution kinetics and limited gastrointestinal absorption. Improving the solubility and dissolution rate of cilostazol is essential to achieve predictable bioavailability and consistent therapeutic efficacy [14].

Recent advances have explored the potential of nanoformulations of cilostazol to enhance its bioavailability, targeted delivery, and therapeutic efficacy [15]. Preliminary studies suggested that Nano-Cilostazol can significantly mitigate oxidative damage, suppress pro-inflammatory cytokines, and prevent cellular apoptosis [16,17]. Therefore, Nano-Cilostazol holds potential as a novel therapeutic strategy to prevent or attenuate AKI caused by CIS, addressing the unmet need for effective nephroprotective interventions.

Spanlastics are elastic and deformable surfactant-based nano-vesicles that serve as versatile drug delivery systems for a broad spectrum of therapeutic agents. These nanostructures are formed through the self-assembly of non-ionic surfactants and edge activators in aqueous media. Their biodegradable, biocompatible, and non-immunogenic properties make them an attractive platform for enhancing drug delivery [18,19].

Therefore, the purpose of our investigation was to assess Nano-Cilostazol’s ability to prevent CIS nephrotoxicity. We also attempted to clarify underlying pathways in the roles of ASC, NLRP3, GSDMD, JAK2, STAT3, and miRNA-155.

## 2. Materials and Methods

### 2.1. Chemicals

Sorbitan monostearate (Span 80) (Oxford laboratory, Mumbai, India), Tween 80 (El Naser pharmaceutical chemicals, Cairo, Egypt), Pluronic F-127 (BASF Corporation, Florham Park, NJ, USA), and chloroform were used. The kits utilized for biochemical analysis were procured from the Biodiagnostics Company located in Dokki, Giza, Egypt. All utilized reagents were of high analytical grade.

### 2.2. Preparation and Characterization of Nano-Cilostazol

#### 2.2.1. Preparation of Nano-Cilostazol

The Nano-Cilostazol was prepared by a slight modification of a previously published method for the preparation of spanlastic nanoparticles [20,21,22,23,24]. In brief, Cilostazol (100 mg) was dissolved in the least amount of chloroform (2 mL) and it was mixed with span 80 (2 g). The mixture was then heated to 70 °C at 300 rpm with the aid of a hot plate (Stuart, Caliber Scientific, Holland, OH, USA). Tween 80 (0.5 g) and Pluronic F-127 (0.25 g) were dissolved in 50 mL of deionized water with the aid of a hot plate (the temperature was adjusted to 70 °C). The molten cilostazol/span 80 mixture was transferred into the tween 80 solution with continuous stirring (400 rpm/70 °C), the mixture was equilibrated for 30 min, followed by treatment with probe sonication (Sonic Vibra Cell, Newtown, CT, USA), (15 s pulse and 10 s pause) at 50% amplitude (130 W) for 5 min.

#### 2.2.2. Characterization of Nano-Cilostazol

Zeta potential (ζ), particle size (PS) and Polydispersity index (PDI) of Nano-Cilostazol.

The zeta potential, particle size, and polydispersity index for the prepared nano-vesicles were estimated with the aid of a zetasizer (Malvern Instruments, Malvern, UK). Three samples were suitably diluted with deionized water and stabilized for 15 min at 25 °C to facilitate measurement in optimal conditions. The results were expressed as mean ± standard deviation (SD) [25].

***Transmission electron microscope (TEM).***

The exact particle size and shape of Nano-Cilostazol was investigated with the aid of a transmission electron microscope (TEM, JEM-2100F, electron microscope, JEOL Ltd., Tokyo, Japan). The colloidal dispersion was suitably diluted and loaded onto a membrane-coated grid surface before examination for 2 min. Excess liquid was removed with filter paper [23].

***FTIR analysis.***

Fourier-transform infrared spectroscopy (FTIR; BRUKER, Billerica, MA, USA) equipped with an air-cooled deuterated triglycine sulfate (DTGS) detector was employed to identify functional groups of Cilostazol and its corresponding nano-formulation. The samples were compressed into a potassium bromide (KBr) pellet using a hydraulic press. Spectral analysis was performed over a wavenumber range of 400–4000 cm^−1^.

### 2.3. Molecular Docking

#### 2.3.1. Ligand Preparation

The ligands employed in this study, cilostazol (CID: 2754) and Disulfiram (CID: 3117) (as a reference ligand), were retrieved from PubChem. Then, energy minimization was performed using the MMFF94 [26] force field implemented in Avogadro 1.2.0 [27] software to obtain optimized conformations of the ligands.

#### 2.3.2. Protein Preparation

Disulfiram was selected as a reference compound due to its reported inhibitory effects on inflammasome-mediated oxidative stress and regulated cell death pathways. Although disulfiram primarily acts through the thiol-reactive inhibition mechanism, it is an FDA-approved drug with documented ability to modulate key downstream processes, including ASC recruitment and GSDMD-mediated pyroptosis [28], as well as upstream suppression of NLRP3 inflammasome activation via modulation of NLRP3 palmitoylations [29]. Beyond pyroptosis, disulfiram has demonstrated regulatory effects on microRNA expression and the JAK2/STAT pathway, along with cytoprotective effects on KIM-1 and BAX expression [30,31]. Owing to this broad mechanistic overlap with the investigated molecular targets, disulfiram serves as a robust benchmark for comparative docking analyses with cilostazol.

For the docking simulations, rat-specific protein sequences for BAX (Q63690), ASC (p63116), GSDMD (A0A096MJ11), KIM_1 (o54947), JAK2 (Q62689), and NLRP3 (D4A523), along with the sequence of miRNA-155, were obtained from the Uniprot database.

As experimental crystal structures were unavailable for the investigated targets, protein structures were obtained from the Alpha-fold protein structure database to ensure the use of complete three-dimensional models without missing atoms. All structures were prepared for docking using Auto Dock Tools 1.5.7 [32], which involved the removal of water molecules, addition of polar hydrogen atoms, and assignment of Gasteiger charges to ensure computational accuracy and consistency across docking simulations [33].

#### 2.3.3. Active Site Prediction and Molecular Docking

Binding sites of individual proteins were initially identified through a literature review and subsequently verified and predicted using the CB-DOCK2 blind docking server [34] and uniport formation, which employs a cavity detection algorithm to predict likely ligand-binding sites. Molecular docking simulations were performed using QuickVina-2 (QVina2) software [35]. The predicted template modeling (pTM) scores for the AlphaFold models were 0.41 for miRNA-155, 0.76 for GSDMD, 0.78 for ASC, and 0.45 for KIM-1. For protein with previously reported functional regions (which were retrieved using UniProt IDS), binding site selection was guided by known key residues, including JAK2 (residue 976) and NLRP3 (residue 520). In addition, the AlphaFold-predicted BAX model was analyzed using CB-DOCK2 and exhibited a high average pLDDT score (85.94), indicating good structural reliability for docking analysis.

### 2.4. Experimental Animals

Forty male albino Wistar rats weighing between 150 and 180 g were provided with water and regular meals ad libitum and maintained for two weeks to acclimate prior to the experiment under prescribed conditions (12 h light/dark cycle, temperature 22 ± 3 °C, and fifty percent humidity). The study was approved in accordance with the Institutional Animal Care and Use procedures of the Faculty of Veterinary Medicine, Alexandria University, Egypt. The Faculty of Veterinary Medicine’s Research Ethical Committee, Alexandria University, Alexandria, Egypt, approved the experimental procedures (ALEXU-IACUC, 013-2025-12-05/353) (12 May 2025). Every technique was carried out in compliance with the applicable rules and regulations. All methods adhered to the ARRIVE standards [36].

### 2.5. Experimental Protocol 

**Figure 1:**
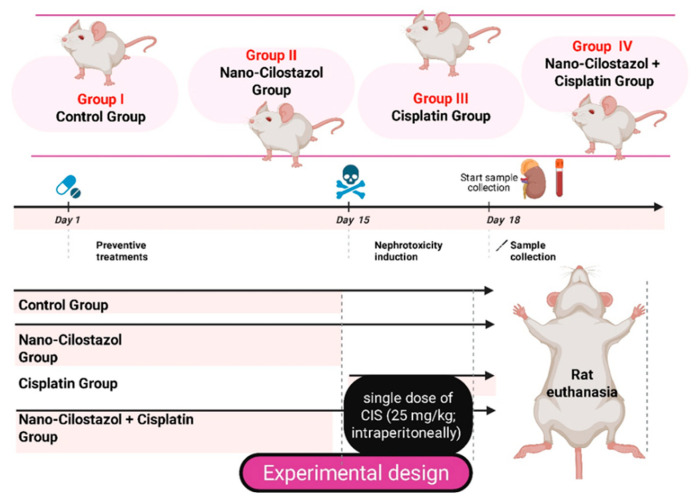
Experimental timeline and design.

Forty male adult albino rats were allocated into four groups (*n* = 10/group) (Figure 1). Group I (Control) received normal saline. Group II (Nano-Cilostazol) received a daily oral dose of Nano-Cilostazol (10 mg/kg) for 15 days [16]. Group III (CIS) received a single dose of CIS (25 mg/kg; intraperitoneally) on day 15 [37]. Group IV (Nano-Cilostazol + CIS) received Nano-Cilostazol at the previously mentioned dose for 15 consecutive days and was then intraperitoneally injected with a single dose of CIS on the 15th day. After 72 h, the rats were sacrificed.

### 2.6. Blood and Tissue Sampling

After euthanasia with isoflurane, samples of blood were obtained from the aorta in plain tubes and then left to coagulate for 30 min at the slop position. Blood tubes were centrifuged for 15 min; the serum was apportioned into clean Eppendorf tubes for further biochemical studies. The kidneys of all animals were cautiously dissected; the right kidneys were fixed for histopathological and immunohistochemical analysis in 10% neutral buffered formalin; the left kidneys were washed in saline for homogenization and kept at −80 for further biochemical and molecular analyses.

### 2.7. Serum Biochemical Indicators of Renal Function

The serum urea and creatinine levels were measured according to Patton and Crouch [38] and Bowers and Wong [39], respectively. Quantitative determination of serum Cystatin-c, Neutrophil Gelatinase Associated Lipocalin (NGAL) and C-reactive protein (CRP) was carried out using Rat ELISA Kits (CSB-E08385r., Biospes (Chongqing, China), BEK1168., and CSB-E07922r., respectively). Every biochemical test was evaluated following the manufacturer’s instructions.

### 2.8. Evaluation of Renal Pro-Inflammatory Cytokines

Tumor necrosis factor–α (TNF-α) and interleukin-1β (IL-1β) were quantitatively assessed in kidney tissue using rat-specific ELISA kits (BEK1214 and BEK1096, respectively) by following the manufacturer’s guidelines.

### 2.9. Assessment of Apoptosis and Pyroptosis Biomarkers in Renal Tissues by ELISA

Apoptosis regulator BAX (BAX) and B-cell CLL/lymphoma 2 (BCL2) were quantitatively determined in renal homogenate using a rat-specific ELISA Kit. Likewise, for the quantitative assessment of Caspase-1 in renal homogenate, Rat Caspase-1 (CASP1) ELISA Kit was used. Rat ELISA kits for BAX, Bcl2, and Caspase-1 (EL002573RA, CSB-E08854r, and CSB-EL004543RA, respectively) were purchased from CUSABIO Co. (Houston, TX, USA). All parameters were assayed according to the manufacturer’s instructions.

### 2.10. Evaluation of Renal Oxidative Stress Biomarkers

Malondialdehyde (MDA) was measured in kidney tissue [40]. Superoxide dismutase (SOD) and catalase (CAT) activities were assessed according to the techniques [41,42], respectively. Meanwhile, total glutathione (tGSH), reduced (GSH), and oxidized glutathione (GSSG) contents were estimated [43]. All prior variables were measured spectrophotometrically according to the manufacturer’s directions using commercial kits from Bio-diagnostic Co., Giza, Egypt.

### 2.11. Gene Expression Analysis of KIM-1, ASC, NLRP3, GSDMD, JAK2, STAT3 and MCP-1 Using RT-PCR

Kidney samples were collected and processed as cross sections encompassing the entire renal tissue. For the qPCR assay, total mRNA was extracted from kidney tissue samples using TRIzol reagent (Invitrogen, Carlsbad, CA, USA). The isolated RNA was quantified and checked for quality using a Nanodrop 2000 (Thermo Fisher Scientific, Madison, WI, USA). Reverse transcription was performed, and complementary DNA was synthesized using a Prime Script RT Reagent Kit (Takara Bio, Otsu, Japan) according to the manufacturer’s instructions. The PCR amplification products were quantified by the FastStart Universal SYBR Green Master (Roche, Basel, Switzerland) following a standard procedure (95 °C for 5 s, 55 °C for 15 s and 60 °C for 15 s; 45 cycles). The primer sequences used for NLRP3, ASC (PYCARD), GSDMD, KIM-1, MCP-1, JAK2, STAT3 and 18s rRNA are illustrated in Table 1. The relative fold changes in target gene expression were determined using the comparative 2^−ΔΔCt^ (ct: cycle threshold) method [44].

### 2.12. Relative Quantification of Expression of miR-155 Using PCR

MiR-155 expression in the kidney tissue was assayed using TaqMan^®^ miR-155 (Thermo Fisher Scientific, Waltham, MA, USA, Cat. no. 4427975, ID: 002571) kit and using U6 as a reference gene (cat no. 4427975, ID: 001973). The mature miR-155 sequence is UUAAUGCUAAUUGUGAUAGGGGU. Quantitative PCR began with an initial denaturation at 95 °C for 10 min and amplification via 45 cycles of PCR as follows: Denaturation at 95 °C for 5 s, annealing at 55 °C for 15 s, and then extension at 60 °C for 15 s. Amplification, data acquisition, and analysis were performed on CFX96 Touch Deep Well Real-Time PCR Detection System (Bio-rad laboratories, Hercules, CA, USA). The values of threshold cycle (Ct) were determined using CFX Maestro™ Software version 1.1 (Bio-rad laboratories, Berkeley, CA, USA). The relative changes in miR155 in samples were determined using the 2^−ΔΔCt^ method and normalized to the reference U6 as described previously.

### 2.13. Histopathological Evaluation

After being meticulously dissected, the rats’ kidney samples were immersed in a 10% neutral buffered formalin fixative for a whole day. Following that, the samples underwent histological processing using a standard paraffin embedding method. After that, the samples were cut into 4 µm slices and stained using Mayer’s hematoxylin and eosin (H&E) [45].

Five areas (400 × magnification) from renal slices were chosen at random from each group of rats to provide a semi-quantitative analysis of histopathological lesions. A four-point scale was used to rate the severity of lesions based on the proportion of the affected area or full section. The most significant pathological abnormalities, including vascular, glomerular, and tubular changes, were selected and scored. Histopathological alterations were seen in 0 (no lesion); 1 (mild), less than twenty-five percent; 2 (moderate), from more than twenty-five to fifty percent; 3 (severe), from more than fifty to seventy-five percent, and 4 (extremely severe), from more than seventy-five to one hundred percent of the total fields studied [46].

### 2.14. Immunohistochemical Protein Assay

Using the avidin–biotin–peroxidase method, renal paraffin blocks were sectioned into 4µm thick slices and immunoassayed with nuclear factor kappa B (NF-κB) and caspase-3. To retrieve antigens, renal paraffin slices were first deparaffinized in xylene, rehydrated in decreasing alcohol concentrations, and then microwave-treated (0.01 M Trisodium citrate). Slides were then treated in 10% H_2_O_2_ to deactivate endogenous peroxidase. Mouse monoclonal antibodies, or primary antibodies, used to detect NF-κB (Catalog # (A-12): sc-514451) and caspase-3 (31A1067: sc-56053), were put on slices and then incubated overnight at 4 °C. Antibodies were purchased from Santa Cruz, Dallas, TX, USA. First, a secondary biotinylated antibody was utilized, followed by streptavidin–horseradish peroxidase incubation. Phosphate-buffered saline was added to the slides three times after each stage. Following diaminobenzidine chromogen solution staining, slides were counterstained with Mayer’s hematoxylin [47]. Using FIJI Image J analysis software, version 2.9.0 (National Institutes of Health, Bethesda, MD, USA), the area percentage of brown immunopositivity expression for NF-κB and caspase 3 was estimated in five random fields of each slide (Image J, NIH, Bethesda, MD, USA).

### 2.15. Statistical Analysis

The Statistical Analysis System software (GraphPad Prism, version 7) was used to perform a one-way analysis of variance (ANOVA) on the laboratory data. Tukey’s test was used to assess the differences in means. Non-parametric lesion scoring was analyzed using the Kruskal–Wallis test followed by Dunn’s post hoc test. Mean ± standard error (means ± S.E.) was used to represent the statistical data [48].

## 3. Results

***Nano-Cilostazol characterization.***

**Figure 2:**
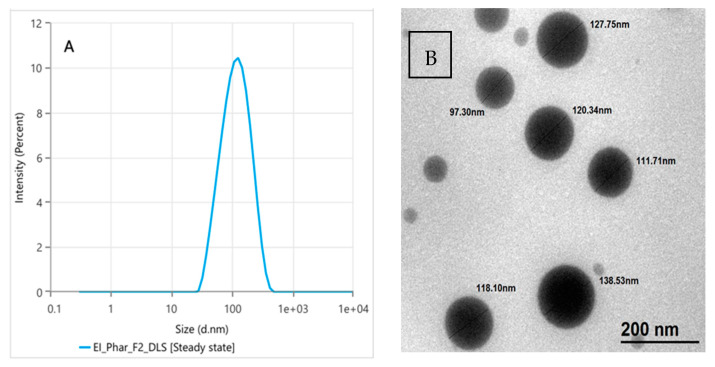
The DLS (**A**) and TEM examination (**B**) of Nano-Cilostazol.

The results of zeta potential, PS, and PDI DLS of Nano-Cilostazol were estimated. The average zeta potential was −37.31 ± 1.56 mV, and the mean PS was 101.07 ± 0.50 nm (Figure 2A), while the PDI was 0.196 ± 0.005. These results were confirmed by TEM examination of the nanoparticle, which revealed dark, smooth, spherical particles with a diameter ranging from 97.3 to 138.53 nm (Figure 2B).

**Figure 3:**
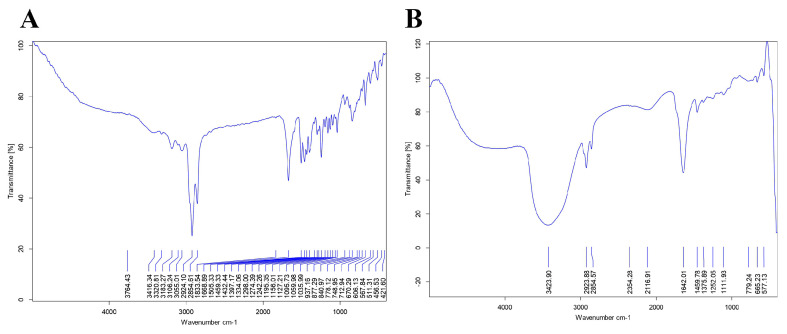
FTIR of Cilostazol (**A**) and Nano-Cilostazol (**B**).

The FTIR of Cilostazol was investigated. The FTIR illustrated the stretching vibration of both aliphatic and aromatic C-H functional groups at 2924 and 2854 cm^−1^ respectively. The presence of a characteristic sharp peak at 1668 cm^−1^ indicated the C=N stretching of the tetrazole group, while the small weak peak at 3320 cm^−1^wasrelated to N-H stretching of quinolinone (Figure 3A).

The FTIR of Nano-Cilostazol was quite similar to the FTIR of Cilostazol, indicated by the presence of two peaks at 2923 and 2845 cm^−1^ and the sharp characteristic peak at 1642 cm^−1^. However, a strong wide peak appeared at 3424 cm^−1^, which can be explained by the formation of hydrogen bonding. Additionally, the peak in the characteristic fingerprint area became less prominent, which may be related to the complete entrapment of cilostazol in the vesicular structure (Figure 3B).

***Molecular docking results.***

Below are the molecular docking binding affinities of Cilostazol and Disulfiram against seven key protein targets involved in acute kidney injury pathophysiology. Binding affinity scores are expressed as ΔG in kcal/mol, where more negative values indicate stronger binding interactions. All docking simulations were performed using QuickVina-2 software, with binding sites predicted by the CB-Dock2 server (Table 2).

A detailed molecular interaction profile of Cilostazol with seven protein targets involved in acute kidney injury. The table presents specific amino acid residues involved in binding, interaction distances in Ångströms (Å), and the types of intermolecular forces (hydrogen bonds, hydrophobic interactions, and electrostatic interactions) that stabilize the protein–ligand complexes. UNL1 denotes the Cilostazol ligand molecule. Interactions were analyzed using standard molecular docking visualization protocols (Table 3).

**Figure 4:**
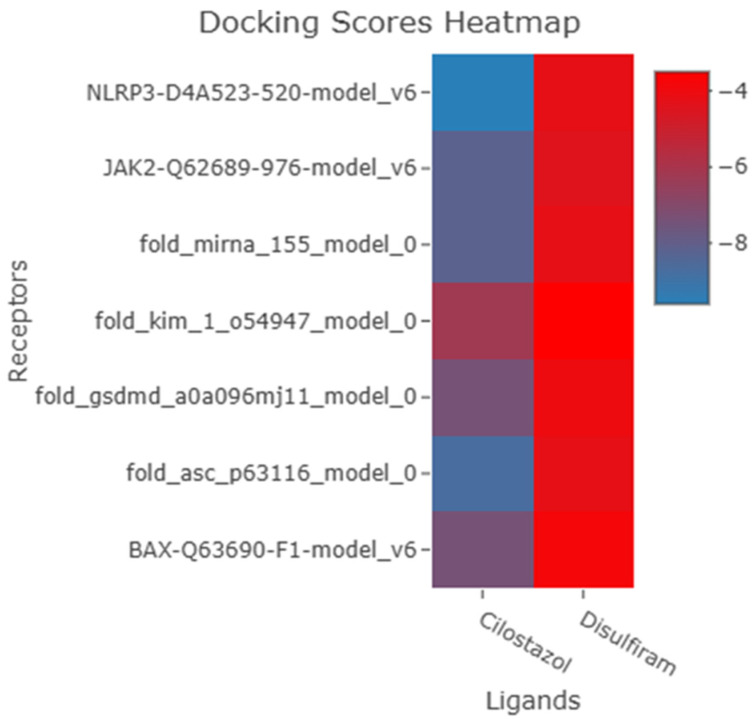
Heatmap visualization using R ggplot for docking results shows the most negative values, which indicate more binding, represented by blue grading shadows. The figure shows the superiority of Cilostazol over disulfiram.

A heatmap visualization using R ggplot for docking results shows the most negative values, which indicate more binding favorability, represented by blue grading shadows. The figure shows the superiority of Cilostazol compared to disulfiram (Figure 4).

**Figure 5:**
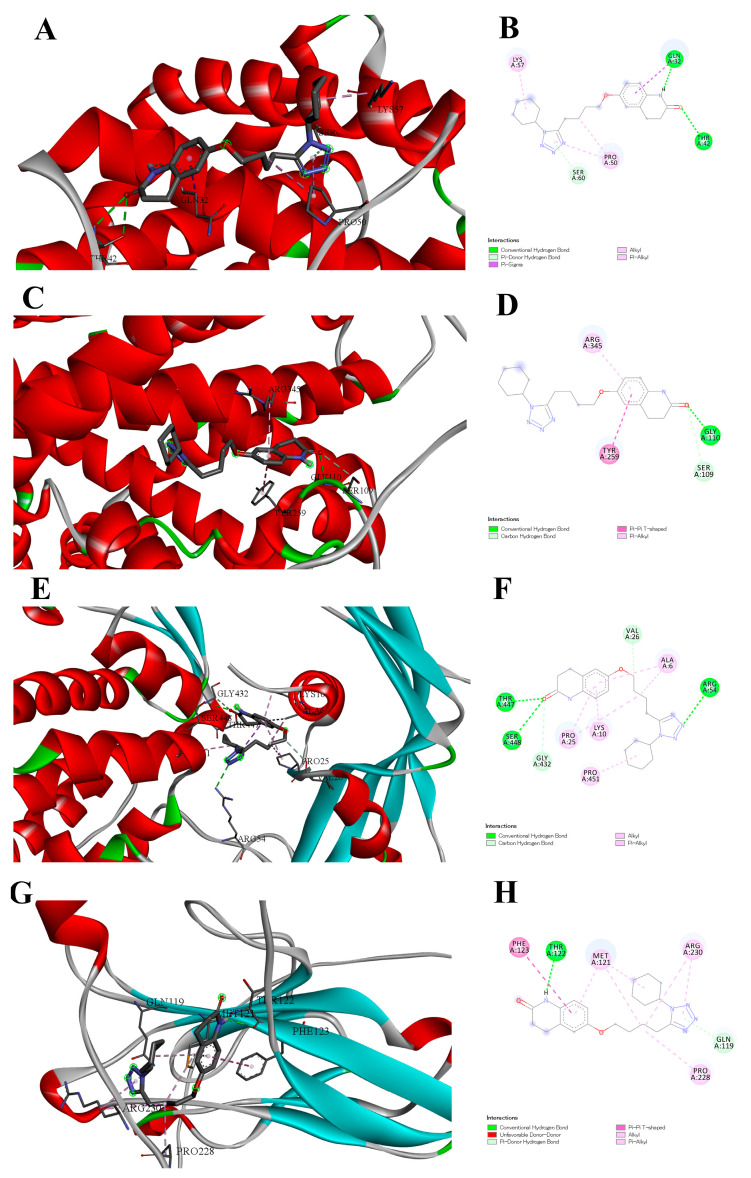
Molecular docking of the interaction of cilostazol with Bcl-2–associated X protein (BAX) (**A**,**B**), apoptosis-associated speck-like protein containing a CARD (ASC) (**C**,**D**), Gasdermin D (GSDMD) (**E**,**F**), and Kidney Injury Molecule-1 (KIM-1) (**G**,**H**). Structural elements of (**A**,**C**,**E**,**G**) are colored as follows: α-helices: red, β-sheets: green and cyan/blue, cyan helical regions: cyan, loops/coils: gray, Ligand (Cilostazol): black stick form, Ligand heteroatoms nitrogen in blue, oxygen in red, Hydrogen bonds: dashed lines.

The following are depicted in Figure 5: Figure 5A depicts a 3D visualization of interaction between Cilostazol and the BAX protein using Biovia 2020. The figure shows a binding interaction inside the red alpha helix by targeting GLN, SER and LYS amino acids. Figure 5B shows a 2D visualization of the interaction between Cilostazol and the BAX protein using Biovia 2020; the figure shows binding interactions within the pocket by targeting GLN32, SER60, and LYS57 amino acids. Figure 5C shows a 3D visualization of the interaction between Cilostazol and the ASC protein using Biovia 2020; the figure shows a binding interaction inside the red alpha helix by targeting ARG, SER and GLY amino acids. Figure 5D shows a 2D visualization of the interaction between Cilostazol and the ASC protein using Biovia 2020; the figure shows a binding interaction inside the pocket by targeting ARG345, TYR259, SER109 and GLY110 amino acids. Figure 5E shows a 3D visualization of the interaction between Cilostazol and the GSDMD protein using Biovia 2020; the figure shows a binding interaction inside the red alpha helix and blue B-sheets by targeting PRO, SER and GL Yamino acids. Figure 5F shows a 2D visualization of the interaction between Cilostazol and the GSDMD protein using Biovia 2020; the figure shows a binding interaction inside the red alpha helix and blue B-sheets by targeting THR447, PRO 25 and 451, SER448 and GLY432amino acids. Figure 5G shows a 3D visualization of interaction between Cilostazol and the KIM-1 protein using Biovia 2020;the figure shows a binding interaction within structural elements including cyan helical regions, with the ligand positioned in the binding pocket interacting with GLN, THR, MET, PHE, ARG, and PRO amino acids. Figure 5H shows a 2D visualization of the interaction between Cilostazol and the KIM-1 protein using Biovia 2020; the figure shows a binding interaction inside the pocket through hydrogen bonding with THR and GLN residues, pi–pi T-shaped interaction with PHE, and multiple hydrophobic contacts with MET, ARG, and PRO amino acids.

**Figure 6:**
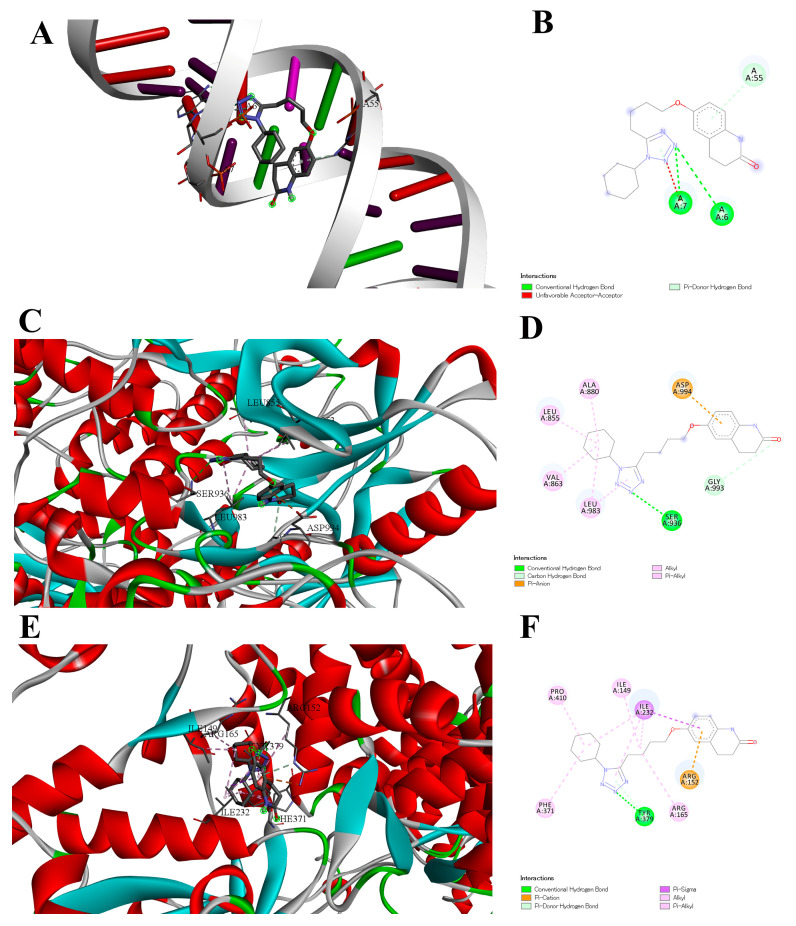
Molecular docking of the interaction of cilostazol with miRNA-155 (**A**,**B**), JAK2 (**C**,**D**) and NLRP3 (NOD-, LRR-, and pyrin domain-containing protein 3) (**E**,**F**). Structural elements of (**A**,**C**,**E**) are colored as follows: α-helices: red, β-sheets: green and cyan/blue, cyan helical regions: cyan, loops/coils: gray, Ligand (Cilostazol): black stick form, Ligand heteroatoms nitrogen in blue, oxygen in red, Hydrogen bonds: dashed lines.

In terms of Figure 6, Figure 6A shows a 3D interaction between miRNA_155 and Cilostazol, with specific interaction with adenine nitrogen bases (shown in red). Figure 6B shows 2D interaction plot for Cilostazol with adenine bases, which is favorable with H-Bonds and pi-Donor H-Bonds. Figure 6C depicts a3D visualization of the interaction between Cilostazol and the JAK2 protein using Biovia 2020; the figure shows a binding interaction between red alpha helices and cyan structural regions, with the ligand engaging with SER, LEU, and ASP amino acids within the kinase binding pocket. Figure 6D shows a 2D visualization of the interaction between Cilostazol and the JAK2 protein using Biovia 2020; the figure shows a binding interaction inside the pocket featuring conventional hydrogen bonding with SER, a carbon–hydrogen bond with GLY, pi–anion interaction with ASP, and extensive hydrophobic contacts with LEU, VAL, and ALA amino acids. Figure 6E depicts a 3D visualization of the interaction between Cilostazol and the NLRP3 protein using Biovia 2020;the figure shows the binding interaction within a deep pocket surrounded by red alpha helices and cyan beta sheets, targeting ARG, ILE, TYR, and PHE amino acids. Figure 6F shows a 2D visualization of the interaction between Cilostazol and the NLRP3 protein using Biovia 2020; the figure shows a binding interaction inside the pocket through conventional hydrogen bonding with TYR, pi–cation and pi–donor hydrogen bonds with ARG, pi–sigma interaction with ILE, and multiple hydrophobic contacts with PRO, ILE, ARG, and PHE amino acids.

***Effect of Nano-Cilostazol on changes in renal function indices induced by CIS toxicity in male rats.***

**Figure 7:**
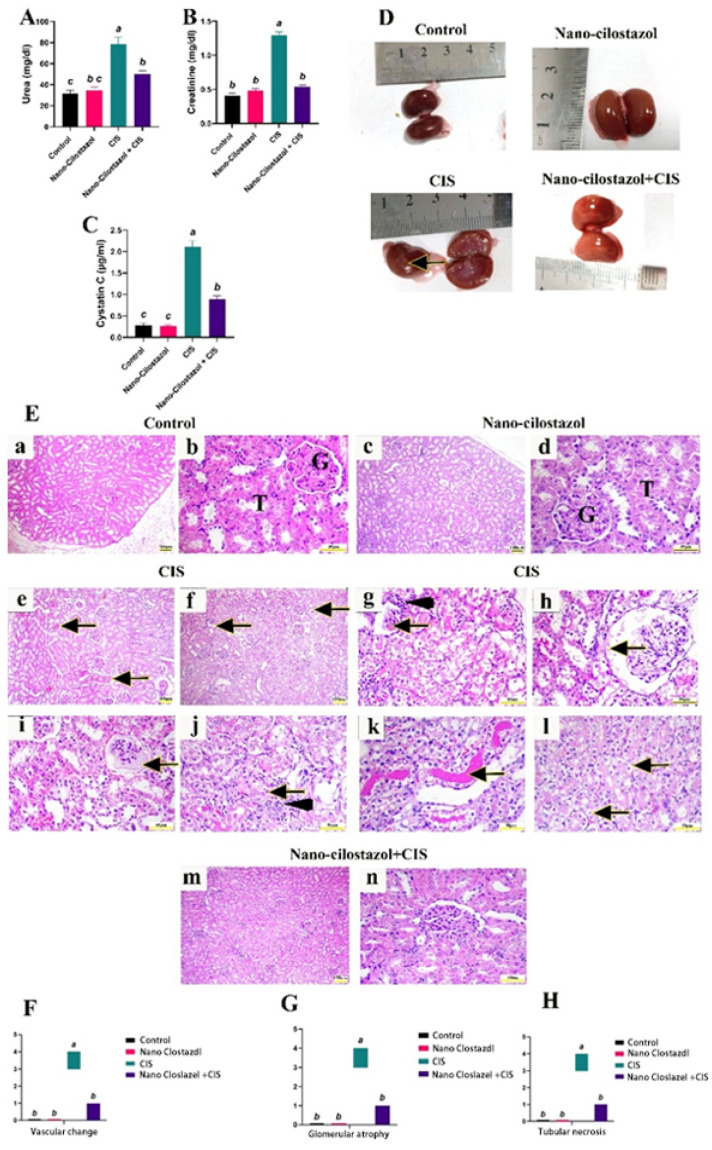
Effects of Nano-Cilostazol on renal (**A**) urea (mg/dL), (**B**) creatinine (mg/dL), and (**C**) cystatin-C (µg/mL). (**D**) Gross pictures from different experimental groups (arrow indicates white necrotic areas). (**E**) Histopathologic changes from different experimental groups. (**a**–**d**) Control and Nano-Cilostazol groups showing normal renal histopathologic picture with typical glomerular (G) and tubular (T) structure, (**e**–**i**) Cisplatin treated group showing (**e**) Congested vessels (arrows), (**f**,**g**) Glomerular atrophy (arrows) with mononuclear infiltrates (arrowhead), (**h**,**i**) Widening of Bowman’s capsule and faint eosinophilic glomerular infiltrates (arrows), (**j**) Tubular necrosis with mononuclear infiltrates (arrowhead) and apoptotic bodies (arrow), (**k**) Hyaline cast formation (arrow), (**l**) Tubular epithelial vacuolization (arrows). (**m**,**n**) Combination group showing normal renal histoarchitecture. (**F**–**H**) Semiquantitative lesion analysis of vascular change, glomerular atrophy and tubular necrosis. Scale Bar (**a**,**c**,**e**,**f**,**m**) = 100 µm and Scale Bar (**b**,**d**,**g**–**l**,**n**) = 50 µm. Bars bearing different letters were substantially different at *p* < 0.05. Results are presented as means with SE.

Figure 7A–C report that CIS administration exhibited a significant elevation in serum renal urea, creatinine, and cystatin-C levels (by 147%, 214%, and 654%, respectively) compared to the control group. However, the co-treatment with Nano-Cilostazol led to a significant reduction in these parameters by 36.04%, 58.3% and 57.9%, respectively, compared to the CIS-treated group. No important alterations were reported between control and Nano-Cilostazol-treated rats in the abovementioned measurements.

***Effect of Nano-Cilostazol on the kidney gross picture induced by CIS toxicity in male rats.***

Both control and Nano-Cilostazol-treated groups exhibited brown kidneys with a smooth surface. On the other hand, CIS-intoxicated rats revealed pale kidneys with multiple white areas indicating necrosis. However, Nano-Cilostazol with the CIS-treated group showed pale kidneys without any necrotic changes (Figure 7D).

***Effect of Nano-Cilostazol on histopathological changes induced by CIS toxicity in male rats.***

Both control (Figure 7(Ea,Eb)) and Nano-Cilostazol-treated (Figure 7(Ec,Ed)) groups exhibited a typical structure of the glomerulus, renal corpuscle, proximal and distal convoluted tubules. On the other hand, CIS-intoxicated rats revealed marked congestion of large blood vessels, inter-tubular blood capillaries, and glomerular tufts Figure 7(Ee)), besides glomerular shrinkage and atrophy (Figure 7(Ef,Eg)), a widening of Bowman’s capsule and faint eosinophilic glomerular infiltrates (Figure 7(Eh,Ei)). Most renal tubules had extensive coagulative necrosis with mononuclear inflammatory infiltrates, with the presence of some apoptotic bodies (Figure 7(Ej)), while other renal tubules had eosinophilic intra-tubular infiltrates in their lumen (Figure 7(Ek)) with severe epithelial vacuolization (Figure 7(El)). However, Nano-Cilostazol with the CIS-treated group showed marked improvement for renal histo-architecture, limited to only mild interstitial congestion of inter-tubular blood capillaries and mild epithelial degeneration of tubules with restoration of glomerular tuft size (Figure 7(Em,En)). The kidney damage scores of the control and Nano-Cilostazol-treated animals did not vary significantly. In comparison to the control animals, the CIS-treated group showed a remarkable increment (*p*  <  0.05) in vascular, glomerular, and tubular injury scores. However, rats treated with Nano-Cilostazol and CIS showed a significant decrease (*p*  <  0.05) in damage scores when compared to the CIS-treated group (Figure 7F–H).

***Effect of Nano-Cilostazol on changes in inflammation induced by CIS toxicity in male rats.***

**Figure 8:**
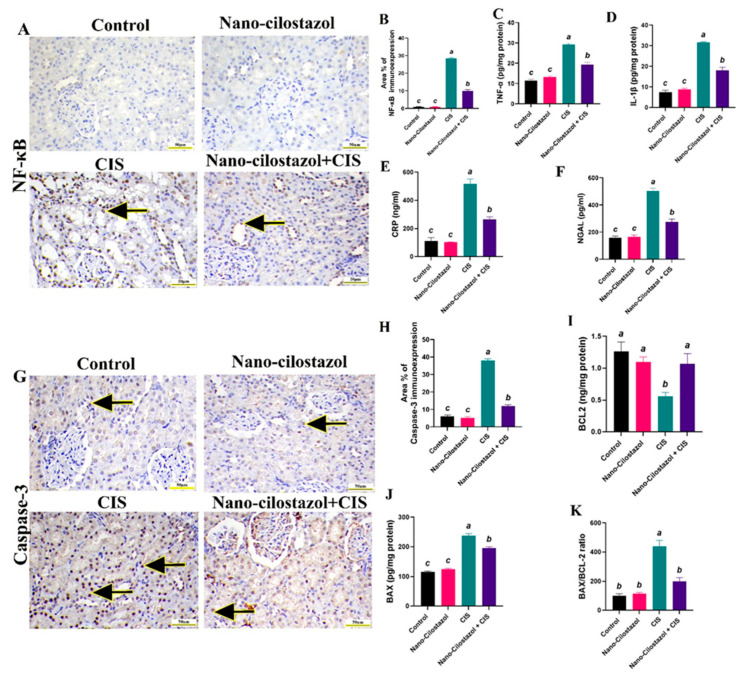
Effects of Nano-Cilostazol on renal (**A**) nuclear factor kappa B (NF-κB) immunoexpression in different groups (Scale bar = 50 µm). (**B**) Area % of NF-κB immunostaining. (**C**) TNF-α (Tumor necrosis factor alpha, pg/mg protein). (**D**) Il-1β (Interleukin-1β, pg/mg protein). (**E**) CRP (C-reactive protein, ng/mL). (**F**) NGAL (Neutrophil Gelatinase-associated Lipocalin, pg/mL). (**G**) Caspase-3 immunoexpression in different groups (Scale bar = 50 µm).(**H**) Area% of caspase-3 immunoreactivity. (**I**) BCL2 (B-cell lymphoma 2, ng/mg). (**J**) BAX (BCL-2-associated X protein, ng/mg) and (**K**) BAX/BCL2 ratio. CIS = Cisplatin. Arrows indicate positive nuclear brown immunostaining for NF-κB and caspase-3. Bars bearing different letters were substantially different at *p* < 0.05. Results are presented as means with SE.

Figure 8A showed the immunohistochemical expression of NF-κB. Negative nuclear brown staining for the NF-κB protein was detected in the renal tubular epithelial cells of control and Nano-Cilostazol-treated groups. CIS-injected rats revealed intense nuclear immunoreactivity, while the Nano-Cilostazol plus CIS group showed weak nuclear brown immunostaining in tubular epithelial cells. Statistical analysis of NF-κB immunoreactivity area% showed that no significant differences were detected between control and Nano-Cilostazol-treated groups, while a remarkable three-fold increment was recorded in CIS-intoxicated rats in comparison with the control. Additionally, Nano-Cilostazol plus CIS substantially decreased NF-κB immunoexpression by 64.7% relative to the CIS-only group (Figure 8B).

The data presented in Figure 8C,D revealed a remarkable elevation after CIS injection relative to control data in levels of renal pro-inflammatory cytokines, TNF-α and IL-1β, by 158% and 326%, respectively, indicating an increase in renal inflammatory status. In the Nano-Cilostazol plus CIS group, these elevations were reversed by a significant reduction by 33.9% and 42.8%, respectively, relative to CIS-treated rats. No major variances were reported between control and Nano-Cilostazol-treated rats in the abovementioned parameters.

Similarly, CIS-intoxicated rats exhibited a noteworthy upsurge in serum levels of CRP and NGAL by 335% and 216%, respectively, relative to control rats. These elevations were significantly decreased in the Nano-Cilostazol plus CIS group by 48.8% and 45.1%, respectively, compared to the CIS group. No substantial differences were reported between the control and Nano-Cilostazol-treated rats in the abovementioned parameters (Figure 8E,F).

***Effect of Nano-Cilostazol on apoptotic changes induced by CIS toxicity in male rats.***

Figure 8G illustrates the immunohistochemical expression of caspase-3. Mild nuclear brown staining for caspase-3 protein was detected in the kidney tubules of the control and Nano-Cilostazol-treated groups. In CIS-injected rats, the majority showed intense positive brown immunostaining, while the Nano-Cilostazol plus CIS group showed moderate to weak nuclear brown immunostaining in the tubular epithelium. Statistical analysis of caspase-3 immunoreactivity area % showed no significant differences detected between control and Nano-Cilostazol-treated groups, while a remarkable five-fold upregulation was recorded in CIS-intoxicated rats in comparison with control. Additionally, Nano-Cilostazol plus CIS substantially decreased caspase-3 immunoexpression by 68.9% compared to the CIS-only group (Figure 8H).

Likewise, the CIS group exhibited significantly decreased protein levels of anti-apoptotic BCL-2 by 55.4% and increased levels of pro-apoptotic BAX and the BAX/BCl2 ratio by 103% and 341%, respectively, relative to the control data. Meanwhile, the Nano-Cilostazol + CIS group showed a substantial upregulation of anti-apoptotic BCL-2 by 90.1% and downregulation in levels of BAX and the BAX/BCl2 ratio by 17.6% and 54.8%, respectively, compared to the CIS group. No noteworthy alterations were reported between control and Nano-Cilostazol-treated rats in the abovementioned parameters (Figure 8I–K).

***Effect of Nano-Cilostazol on alterations of oxidative stress and antioxidant biomarkers induced by CIS toxicity in male rats.***

**Figure 9:**
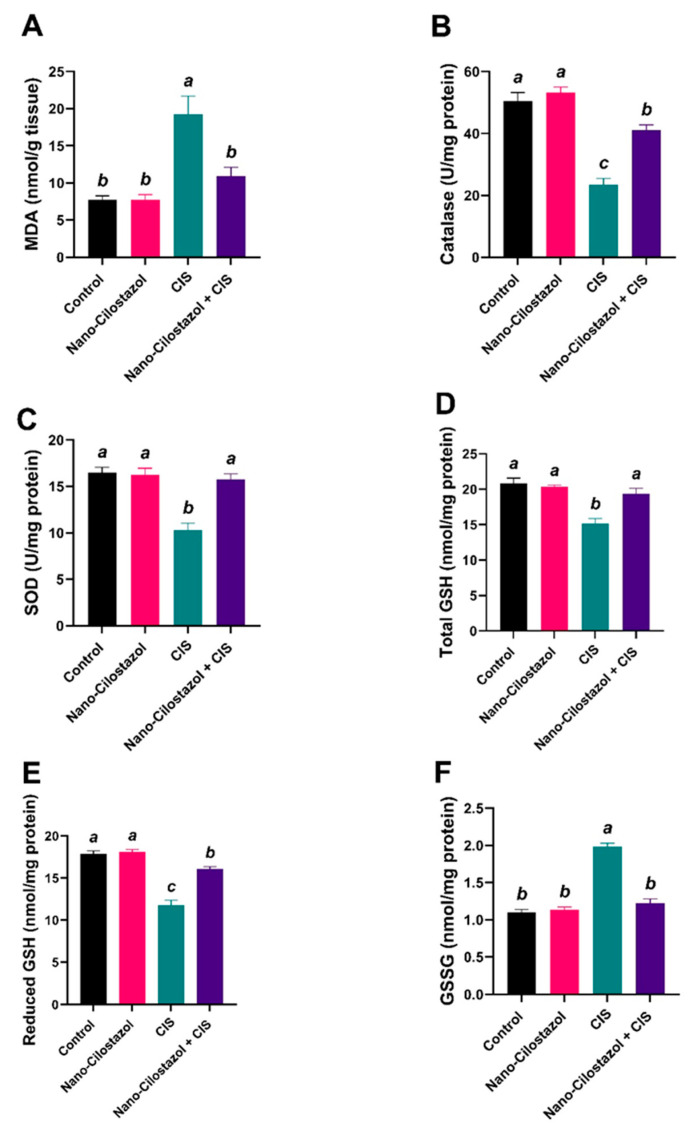
Effects of Nano-Cilostazol on renal (**A**) Malondialdehyde (MDA, nmol/g tissue), (**B**) catalase (U/mg protein), (**C**) SOD (Superoxide dismutase activity, U/mg protein), (**D**) total GSH (total glutathione, nmol/mg protein), (**E**) reduced glutathione (reduced GSH, nmol/mg protein), and (**F**) oxidized glutathione (GSSG, nmol/mg protein). Bars bearing different letters were substantially different at *p* < 0.05. Results are presented as means with SE.

The oxidative stress biomarkers (MDA and GSSG) were significantly upregulated by 148% and 80.4%, respectively, in CIS-treated rats matched with the control rats. However, Nano-Cilostazol treatment with the CIS group showed a remarkable downregulation in those parameters by 43.2% and 38.4% relative to rats treated with CIS only (Figure 9A,F). Additionally, the antioxidant indices of catalase, SOD, total GSH, and reduced GSH were substantially downregulated by 53.5%, 37.5%, 27.4% and 34.2%, respectively, in the CIS-group relative to the control group. However, Nano-Cilostazol treatment with the CIS group showed a remarkable upregulation in those measurements by 75.2%, 52.6%, 27.9% and 36.5%, respectively, compared to rats treated with CIS only (Figure 9B–E). No significant variances were reported between control and Nano-Cilostazol-treated rats in the abovementioned parameters.

***Effect of Nano-Cilostazol on renal injury, pyroptosis, and inflammation induced by CIS toxicity in male rats.***

**Figure 10:**
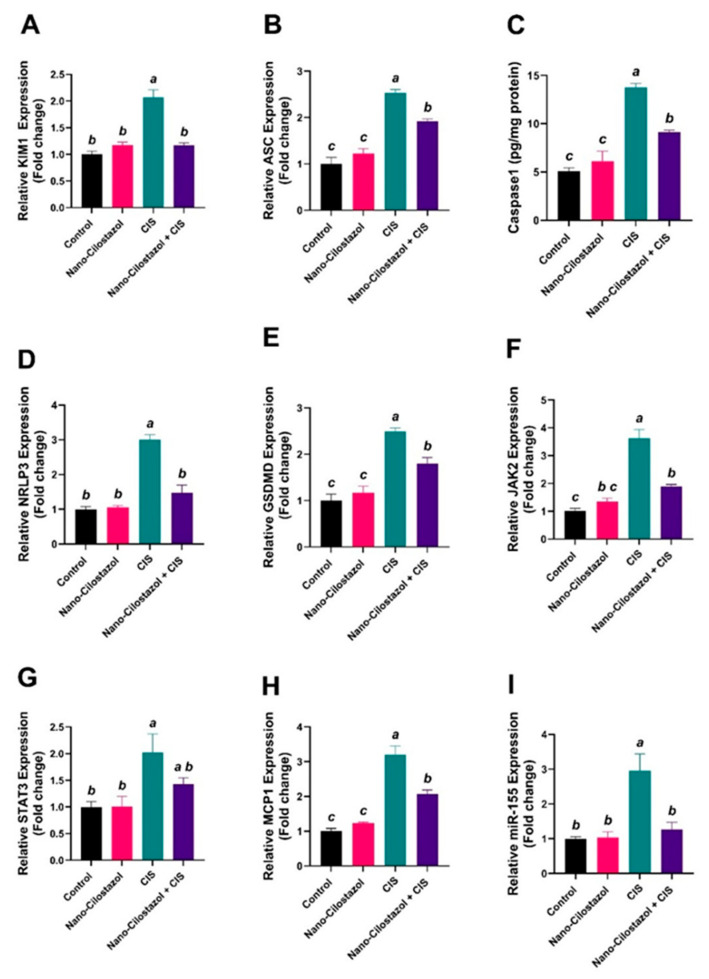
Effects of Nano-Cilostazol on renal (**A**) Relative KIM-1 (Kidney Injury Molecule-1) mRNA transcript expression, (**B**) Relative ASC mRNA transcript expression, (**C**) Caspase-1 protein expression (pg/mg protein), (**D**) Relative NLRP3 (Nod-like pyrin-3) mRNA transcript expression, (**E**) Relative GSDMD (Gasdermin D) mRNA transcript expression, (**F**) Relative JAK2 (Janus kinase 2) mRNA transcript expression, (**G**) Relative STAT3 (Signal Transducer and Activator of Transcription 3) mRNA transcript expression, (**H**) Relative MCP-1 (Monocyte chemoattractant protein-1) mRNA transcript expression and (**I**) Relative miRNA-155mRNA expression. Bars bearing different letters were substantially different at *p* < 0.05. Results are presented as means with SE.

As shown in Figure 10A–H, gene expressions of KIM-1, NLRP3, ASC, GSDMD, JAK2, STAT3, and MCP-1 levels showed a significant upregulation in the abovementioned parameters by 106.7%, 200%, 154%, 149%, 262.7%, 102.3% and 219.7%, respectively, compared to the control group. Nano-Cilostazol reversed the effect of cisplatin on the expression of the previous genes. Nano-Cilostazol treatment in the CIS group indicated a substantial decline in the expression levels of KIM-1, NLRP3, ASC, GSDMD, JAK2 and MCP-1 by 43.5%, 50.8%, 24.2%, 27.7%, 47.9%, and 35.5%, respectively, in comparison with the CIS-only group. However, *STAT3* gene expression revealed an insignificant change in the group treated with Nano-Cilostazol and cisplatin concomitantly when compared to the CIS-treated group. No significant differences were reported between control and Nano-Cilostazol-treated rats in the abovementioned parameters.

***Effect of Nano-Cilostazol on alterations in miRNA-155 induced by CIS toxicity in male rats.***

miRNA-155 expression showed a significant increase by 196% in the CIS-treated group when compared to the control group. Conversely, in the Nano-Cilostazol-andCIS-treated group, miRNA-155 markedly decreased by 57% compared to the CIS-only group. No significant differences were reported between the control and Nano-Cilostazol-treated rats in the abovementioned parameters (Figure 10I).

## 4. Discussion

The current study reported a substantial rise in serum urea and creatinine levels in CIS-intoxicated rats, which is an indication of impaired renal function [49]. Our outcomes are consistent with those of Almutairi et al. [50] and MS Gaballa et al. [51]. This could be explained by CIS-induced decline in the renal blood flow and glomerular filtration rate [52]. Pretreatment with Nano-Cilostazol enhanced the CIS-induced increase in serum levels of urea and creatinine. Our data are consistent with those of Alshahrani et al. [53] and Seleem et al. [54]. This protective effect of Nano-Cilostazol might be attributed to its capacity to lessen oxidative stress and restore mitochondrial dysfunction [55].

Accordingly, serum cystatin-C is also believed to be an early diagnostic indicator of acute kidney injury [56]. Additionally, nephrotoxic damage or renal ischemia typically release a large amount of NGAL into the blood, which is eliminated in the urine [57,58]. Consequently, our results confirmed CIS nephrotoxicity by reporting a noteworthy increase in serum levels of cystatin-C and NGAL in CIS-intoxicated rats. Our results are consistent with those of Ehsan et al. [59], Jana et al. [9], and El-Beltagy et al. [60]. This could be explained by the cytotoxic effect of CIS [59]. On the other hand, the pretreatment of Nano-Cilostazol ameliorated CIS-induced higher serum levels of cystatin-C and NGAL. Similar findings have been reported by Seleem et al. [54]. The renoprotective effect of Nano-Cilostazol may be attributed to its ability to reduce oxidative stress [12].

Our outcomes presented that CIS treatment induced a noteworthy increase in serum CRP, NF-κB immunoexpression and TNF-α and IL-1β in renal tissues. Our findings are in accordance with those of Almutairi et al. [50], Alshahrani et al. [53], Harakeh et al. [61], Mohtadi et al. [62], and Saad et al. [63]. The CIS-induced renal inflammation might be ascribed to the pro-oxidant capacity of CIS to activate the NF-κB pathway [64]. Activated NF-κB trans locates to the nucleus, where it promotes the transcription of TNF-α and IL-1β [65]. TNF-α, the most important cytokine raised during CIS-induced toxicity [66,67], originates from renal epithelial cells and generates ROS. The impaired redox status provokes the immune cells to produce more TNF-α [68]. Oxidative damage and inflammation were associated with the rise in these cytokines [50]. Pretreatment with Nano-Cilostazol ameliorated CIS-induced renal inflammation. The current results have shown a significant decline in the level of serum CRP, NF-κB immunoexpression, and renal TNF-α and IL-1β. Such findings agree with Seleem et al. [54], Wadie et al. [69], and Heeba et al. [70]. This protective effect of Nano-Cilostazol might be attributed to its anti-inflammatory characteristics [13,71]. Moreover, a recent study by Raouf et al. [16] confirmed the potent anti-inflammatory effect of Cilostazol against CIS-induced acute hepatic damage, through reducing the expression of NF-κB and consequently reducing IL-1β and TNF-α.

As a BCS Class II drug, the absorption of cilostazol from the gastrointestinal tract is limited to its dissolution rate, leading to low and variable bioavailability [72]. The spanlastic vesicles can act as a reservoir for Class II drugs, where span 80 reduces the interfacial tension and increases the wettability of the drug and consequently enhances its apparent solubility in gastrointestinal tract fluids [73,74,75,76]. TEM examination illustrated dark spherical vesicles with a particle size of less than 200 nm (the results agreed with the results of our DLS). The nano-sizing of Cilostazol resulted in a massive increase in the surface area to volume ratio, leading to faster dissolution rate according to the Noyes–Whitney equation [77,78,79].

Tween 80 acts as an edge activator, allowing the vesicles to squeeze through pores smaller than their own diameter [79,80]. They can deform and be absorbed via paracellular pathway through the tight junctions in the endothelium of blood capillaries. Additionally, nephrotoxic kidneys have impaired lymphatic drainage and leaky vasculature; consequently, spanlastics can be trapped in the interstitial space of the kidney, leading to passive accumulation of cilostazol at the precise site [81]. Our results illustrated good stability of nano-vesicles, as indicated by the zeta potential (−37.31 ± 1.56 mV) and low values of PDI (0.196 ± 0.005). FTIR showed less prominent peaks at the fingerprint area due to complete sequestration of cilostazol in the vesicular form. It had been molecularly dispersed and incorporated physically into the hydrophobic core of the spanlastic vesicle, which is primarily composed of the non-ionic surfactant [82,83,84].

Our results showed a significant increase in the ratio of BAX/Bcl2 ratio and Caspase-3 in renal tissues of CIS-intoxicated rats. The CIS-induced renal apoptosis may be attributed to the production of ROS, which ultimately results in lipid peroxidation and DNA damage [85,86]. Our findings are in accordance with those of Mohtadi et al. [62], Kim et al. [87], and Aladaileh et al. [88]. Caspase-3 plays a major role in CIS-induced apoptosis [89,90]. This effect results from CIS-induced cellular oxidative stress, increasing the Bax/Bcl2 ratio [91]. Pretreatment with Nano-Cilostazol prevented CIS-induced apoptosis in renal tissues. Results exhibited a noteworthy decrease in the renal BAX/Bcl2 ratio and Caspase-3 in the Nano-Cilostazol group. Our outcomes are in line with those of Raouf et al. [16] and Alshahrani et al. [53]. The anti-apoptotic effect of Nano-Cilostazol may be explained by its capacity to inhibit BAX expression and decrease cytochrome-c release from mitochondria [92].

The current findings reveal the upregulation of NLRP3, ASC, and GSDMD in the cisplatin-treated group. As indicated by our results, cisplatin increases oxidative stress and ROS. Upon recognition of DAMPs, including ROS, NLRP3 recruits a speck-like protein containing a CARD (ASC) and pro-caspase-1 to form the inflammasome. After inflammasome assembly, caspase-1 is activated. Caspase-1 cleaves downstream substrates such as pro-IL-1β, IL-18 and gasdermin D to their mature isoforms. Mature GSDMD perforates the cell membrane, leading to cell swelling and cell death, a process named pyroptosis, accompanied by the leakage of cellular components. The significantly increased levels of caspase-1, along with elevated IL-1β and TNF-α, further confirm inflammasome-mediated inflammatory signaling, contributing to renal tubular damage [93,94]. Li et al. [95] showed that cisplatin activated NLRP3 inflammasome assembly in vivo and in vitro, resulting in the overexpression of cleaved caspase-1, GSDMD, IL-1β and IL-18.

In addition, the upregulation of KIM-1 in the kidney tissue of the cisplatin-treated group serves as a critical indicator of renal tubular injury and can contribute to inflammation and interstitial fibrosis [96,97]. The observed increase in JAK2 and STAT3 expressions in the cisplatin-treated group indicates the involvement of the JAK/STAT signaling pathway in renal tubular apoptosis and ferroptosis. The significant increase in TNF-α in the cisplatin-treated group probably activates the JAK/STAT signaling pathway [98,99]. The results demonstrated by Tsogbadrakh et al. [100] supported these findings, where cisplatin markedly increased the expression of JAK and STAT in NRK-52E normal rat kidney cells. The activation of the JAK2/STAT3 signaling pathway may be related to the overexpression of miRNA-155 in the cisplatin-treated group. miRNA-155 inhibits the expression of Suppressor of Cytokine Signaling 1 (SOCS-1), a negative regulator of the JAK/STAT pathway [11]. The cisplatin-induced inflammatory response includes elevated cytokines and inflammatory cell infiltration in the renal tissue [101,102]. These findings support our results, where cisplatin induced overexpression of MCP-1 in the cisplatin-treated group, resulting in the recruitment of monocytes and macrophages, exacerbating the renal inflammatory response and tissue injury. These results are also consistent with those obtained by Liu et al. [103], where cisplatin significantly elevated the expression of MCP-1 and TNF-α in vitro and in vivo. Lu L. et al. [104] found that macrophage infiltration was doubled in the renal tissue of mice two days after CIS administration. Consequently, targeting MCP1 or its receptors could effectively reduce inflammatory cell infiltration, thereby mitigating significant components of cisplatin-induced renal damage.

The administration of Nano-Cilostazol mitigated cisplatin nephrotoxicity by attenuating inflammatory responses and pyroptosis. Cilostazol acts by inhibitingphosphodiesterase-3, leading to an increase in cyclic adenosine monophosphate (cAMP) levels. Increased levels of cAMP show pleiotropic effects, including anti-inflammatory, antioxidant, and vasodilatory actions [105]. The well-documented effects of cilostazol align with our findings, which demonstrated downregulation of NLRP3, ASC, GSDM, caspase-1, IL-1β, MCP-1, JAK2, and TNF-α. These findings support those of El Awdan et al. [106], who claimed that cilostazol ameliorated thioacetamide-induced liver injury in mice by improving pro-inflammatory cytokines such as TNF-α, IL-1β, NF-kB, and caspase-3. In the same study, cilostazol significantly increased GSH and reduced lipid peroxidation [106]. One possible explanation of the way in which cilostazol suppressed inflammasome assembly is the inhibitory effect of cilostazol on NF-kB [107]. The downregulation of MCP-1 and JAK2 may be attributed to the decreased level of IL-1β, which is crucial for the recruitment of monocytes and macrophages [102]. Perhaps the other most likely explanation is the antioxidant effect of cilostazol. The down-expression of miRNA-155 implies that it exerts a beneficial regulatory effect at the post-transcriptional level, contributing to the overall renoprotective effect by mitigating oxidative stress, pyroptosis, apoptosis and inflammation [108]. Yin et al. [109] demonstrated that inhibition or knockdown of miRNA-155 attenuated pathological damage in acute kidney injury. Chattipakorn et al. [110] found that cilostazol significantly decreased ROS in the heart.

Although there have been major advances regarding the individual molecular targets known to mediate kidney damage, there is still a paucity of effective multi-targeted strategies known to comprehensively modulate the pro-inflammatory state. The proteins BAX, ASC, GSDMD, KIM-1, JAK2, and NLRP3 have all been identified as major perpetrators of renal inflammation and cellular death [111,112]. However, there have still not been any major advances regarding their use as effective dual-targeted strategies for AKI therapy. Additionally, although there have been major advances regarding the pathophysiological importance of miRNA-155 as a mediator of pro-inflammatory responses in several pathologies, there have still been no advances regarding its postulated utility as a direct therapeutic target using small molecules for AKI treatment [113].

The analysis carried out in the current study employed molecular docking simulations so that the binding affinities of Cilostazol and Disulfiram towards the seven key molecular targets that play an essential role in the pathophysiology of AKI could be determined. Based on the findings of our analysis, it has been ensured that the binding affinity of the molecular targets is significantly higher for Cilostazol compared to Disulfiram, ranging from −6.2 to −9.6 kcal/mol in the former, compared to −3.5 to −4.4 kcal/mol in the latter.

The most prominent result came from the Cilostazol–NLRP3 complex, which exhibited the highest binding affinity of −9.6 kcal/mol. The binding analysis of NLRP3 and its ligands depicts a complex binding process that requires both hydrogen bonding and hydrophobic interactions to consolidate the ligands into the NLRP3 binding pocket. The traditional hydrogen bond between TYR379:OH and the nitrogen of Cilostazol at a distance of 2.80 Å depicts a particularly strong polar bond that might contribute to anchoring the ligand into the binding site. This association takes place together with a π–cation bond with ARG152, which might contribute substantial binding free energy to protein–ligand interactions. The biological relevance of such high binding efficiency with NLRP3 cannot be overemphasized since NLRP3 remains as a scaffold protein responsible for activating inflammasomes [94], which might be effectively inhibited to reduce downstream caspase-1 activation with associated processing of pro-inflammatory cytokines IL-1β and IL-18. The presence of a large hydrophobic core network comprising residues ILE232, ILE149, ARG165, and PRO410 might create a hydrophobic microenvironment that would result in high residence time at target locations and could be applicable to anti-inflammatory properties of Cilostazol during clinical practice.

Second, the ASC protein, which plays a crucial role in the functional process of the important adaptor protein that bridges NLRP3 and pro-caspase-1 [114], had the second highest binding affinity for Cilostazol, with a value of −8.7 kcal/mol. It is obvious from the result of the binding process that Cilostazol makes a traditional hydrogen bond to GLY110 and a pi–pi T-shaped bond to TYR259, which is an example of the mentioned aromatic stacking interactions, playing a crucial role in the process of increasing the specificity and strength of the process of binding. At the same time, the discovery of the mentioned type of bonding is highly important in the process of comprehending the mentioned capability of Cilostazol in the binding process to the mentioned hydrophobic pockets of the ASC protein, which plays a highly important role in the process of comprehending the composite protein-protein binding capabilities of the mentioned protein. By the process of binding the mentioned hydrophobic pockets, there is the possibility of steric inhibition of the process of ASC protein binding and aggregation, thus inhibiting the process of inflammasome complex formation in the mentioned important node position. Such an approach, acting on the mentioned NLRP3 and ASC proteins, thus inhibits the formation of inflammasomes in the mentioned important position, and hence, acts on the mentioned two different proteins to develop the mentioned synergistic approach in the process of inhibiting the activation process of inflammasomes by the mentioned multiple and independent processes, thus developing the mentioned possibility of redundancy that could prove beneficial in the process of development of therapeutic strategies in the mentioned context.

The binding affinity exhibited by both JAK2 and miRNA-155, −8.2 kcal/mol, is also quite intriguing. In the case of JAK2, the binding is manifested by standard hydrogen bonding involving SER936, a significant pi-anion interaction involving ASP994, in addition to hydrophobic interaction centered on leucine and valine residues in the binding pocket. JAK2 is known for a crucial role in inflammatory signaling pathways mediated by the JAK-STAT signaling pathway [115], where the overexpression of this enzyme is thought to contribute to the maintenance of inflammation in kidney damage. Based on the binding model, it appears as if the Cilostazol molecule might bind a regulatory region in JAK2, which might alter either the kinase function or the binding of the substrate. However, the carbon-hydrogen bond involving GLY993, in addition to a number of alkyl or pi-alkyl binding modes involving residues LEU855, VAL863, ALA880, and LEU983, provide a binding conformation like other known JAK2 inhibitors, implying a possible usage of Cilostazol in suppressing inflammation by a similar mechanism involving, in part, the modulation of JAK2.

The miRNA-155 interaction, although unusual in that miRNAs are generally regulated transcriptionally/processed rather than by binding small molecules, is an intriguing result. The binding energy of −8.2 kcal/mol and the presence of multiple hydrogen bonds to adenine bases (A6:N6 and A7:N6) indicate that the binding of Cilostazol may occur to the secondary/tertiary structure of this regulatory RNA. The biochemical consequences of small molecules and miRNA binding have been noted as an area of developing research [116], although it would appear plausible that the binding of Cilostazol could inhibit the miRNA-155 interaction with target mRNAs or processing enzymes and mitigate its inflammatory actions. MiRNA-155 has been identified to induce an inflammatory phenotype through the repression of anti-inflammatory mediators, and its expression has been noted in animal models of renal injuries.

The GSDMD protein, responsible for pyroptotic cell death, has a binding affinity of −7.4 kcal/mol to Cilostazol, similar to the binding affinity of BAX. The interaction study indicates conventional hydrogen bonds not only to ARG54, THR447, and SER448 but also a large number of hydrophobic interactions. GSDMD is proteolytically cleaved by inflammatory caspases to produce an N-terminal domain of GSDMD, which oligomerizes and leads to pore formation and subsequently pyroptotic cell death and the consequent release of pro-inflammatory mediators [117]. The interaction of Cilostazol was significant to GSDMD, specifically in the regions involving THR447 and SER448, located close to key regulatory areas. The large number of pi-alkyl interactions involving residues ALA6, LYS10, and PRO25 indicates the ability of Cilostazol to lock GSDMD in a conformation less likely to be activated; this could provide a proof of concept and a mechanistic intervention by which pyroptotic cell death could be limited.

The pro-apoptotic component BAX, a crucial component of the BCL-2 family of proteins, revealed a binding affinity of −7.4 kcal/mol for Cilostazol with interactions focused around residues THR42 and GLN32. The presence of two conventional hydrogen bonds directed at THR42, involving the backbone nitrogen as well as the hydroxyl group of the side chain, in addition to a high-strength hydrogen bond at GLN32 at an extremely short distance of 2.14 Å, is a determinant of a highly favorable binding geometry. The activation of BAX and subsequent oligomerization at the outer mitochondrial membrane followed by cytochrome-c release initiates downstream events of the apoptotic cascade [118]. The binding preference, especially the pi-donor hydrogen bond at SER60 and the nonpolar interactions at PRO50 and LYS57, indicates a potential for Cilostazol interaction with domains participating in BAX activation and membrane transition. This could have implications for modulating both apoptotic and potentially secondary necrosis pathways following kidney damage.

Although it has the least binding affinity for Cilostazol of −6.2 kcal/mol for the target, it can still be regarded as therapeutically relevant for binding events. The receptor, KIM-1, acts both as a biomarker and as an active participant in kidney injuries by being upregulated in the epithelial cells of the proximal tubule following an injury to facilitate the uptake of apoptotic cells and debris [119]. The conventional hydrogen bond component THR122 and pi–donor hydrogen bond component GLN119, along with the pi–pi T-shaped component PHE123, indicated the potential interaction between Cilostazol and KIM-1 that could possibly alter the receptor and/or signal-modulating properties. The exact-T link between the inhibition of the receptor and interaction between Cilostazol and the receptor has broader views beyond being multi-target.

The comparatively lower docking scores obtained for Disulfiram across all investigated targets (−3.5 to −4.4 kcal/mol) suggest a limited propensity for stable non-covalent binding within the selected binding pockets. This observation is consistent with the established pharmacological profile of disulfiram, whose biological activity is largely mediated through enzyme inhibition and thiol-reactive mechanisms rather than high-affinity reversible binding. Accordingly, the present docking results indicate that disulfiram is unlikely to act as a direct high-affinity ligand for the investigated inflammatory mediators under physiological conditions, supporting its use as a comparative reference compound rather than a primary multi-target binder.

The overall result of this docking experiment helps fill the first knowledge gap by illustrating that Cilostazol certainly has a highly versatile multi-target profile that interacts with central mediators of inflammation and/or cell death involved with AKI. In contrast with drugs that target a single pathway, it would appear that Cilostazol might be able to concurrently target the NLRP3 inflammasome complex (through direct protein binding of both NLRP3 and ASC), JAK-STAT-mediated inflammation pathways (through JAK2 protein interaction), pyroptotic forms of cell death (through protein binding of GSDMD), apoptotic pathways (through BAX protein interaction), or even post-transcriptional processing events (through binding of miR-155). Each of these multi-pathways could offer certain potential advantages over current drugs which target a specific pathway [120].

These computational results correlate well with available clinical and experimental data regarding Cilostazol’s therapeutic activities. Cilostazol is currently recognized for its clinical application as a phosphodiesterase 3 inhibitor, possessing antiplatelet and vasodilatory activities, but its increasing pleiotropic anti-inflammatory and cytoprotective activities, which clearly exceed its established pharmacological mode of action, are also being documented [121]. Previous experimental studies have indicated that Cilostazol possesses anti-inflammatory activity by decreasing production levels of pro-inflammatory cytokines, antioxidant activity by scavenging oxidative stresses, and cytoprotective activity against tissue injury in experimental models, but the molecular mechanisms for its pleiotropic activities have not been clearly elucidated. In light of these previous experimental observations, the results of the present docking study now offer mechanistic insights into Cilostazol’s multiple beneficial activities at the molecular level by directly interacting with multiple players of its pleiotropic biological pathways. The high-affinity docking interactions, especially for NLRP3, ASC, and JAK2, indicate that these interactions are likely possible at pharmacologically efficacious Cilostazol concentration levels, which provides further experimental evidence for Cilostazol’s multiple-target modulation-based advantageous pharmacological activity.

A limitation of this study is that renal cortical cAMP levels were not measured, despite cilostazol’s known ability to increase intracellular cAMP in proximal tubular cells. Therefore, we cannot definitively determine whether the observed renoprotective effects are directly mediated through cAMP-dependent signaling pathways. Also, long-term renal outcomes, including the transition from AKI to chronic kidney disease and persistent electrolyte disturbances such as hypomagnesemia, were not assessed. These chronic consequences are clinically important and warrant further investigation in future studies with extended follow-up periods. Additionally, this design was intended to evaluate the preventive potential of N-C under controlled conditions rather than to directly simulate a clinical regimen. We acknowledge that shorter pretreatment durations may be more clinically practical; however, evaluating abbreviated schedules (e.g., 5-day pretreatment) was beyond the scope of the present study and represents an important direction for future research. Regarding safety, no overt adverse effects or behavioral abnormalities were observed in the treated animals at the administered dose, supporting the tolerability of N-C in this experimental context.

## 5. Conclusions

The current study demonstrates a protective effect of Nano-Cilostazol in halting the progression of cisplatin-induced renal injury in rats, potentially by counteracting the inflammatory, oxidative and apoptotic pathways. Nano-Cilostazol presented a significantly better renal biochemical profile, preserved kidney structure, hampered renal inflammation and tubular injury and reduced apoptosis. The observed results offer a solid basis for future clinical research exploring the benefits of the administration of Nano-Cilostazol prior to the potent chemotherapy cisplatin in the prevention of nephrotoxicity.

## Figures and Tables

**Table 1:** Primers used for the qRT-PCR amplification.

Gene	Accession Number	Primer Sequence	
*KIM-1*	NM_173149.2	**F**	5′ TGGCACTGTGACATCCTCAGA 3′
		**R**	5′ GCAACGGACATGCCAACATA 3′
*NLRP3*	NM_001191642.1	**F**	5′ GTCCAGTGTGTTTTCCCAGAC 3′
		**R**	5′ TTGAGAAGAGACCTCGGCAG 3′
*ASC*	NM_172322.2	**F**	5′ CTGCTCAGAGTACAGCCAGAAC 3′
		**R**	5′ CTGTCCTTCAGTCAGCACACTG 3′
*GSDMD*	NM_001400994.1	**F**	5′ CCAGCATGGAAGCCTTAGAG 3′
		**R**	5′ CAGAGTCGAGCACCAGACAC 3′
*JAK2*	NM_031514.1	**F**	5′ TACTTCCTGACCTTTGCCGT 3′
		**R**	5′ TGATACTGTCTGAGCGCACA 3′
*STAT3*	NM_001430046.1	**F**	5′ AGGAGTCTAACAACGGCAGCCT 3′
		**R**	5′ GTGGTACACCTCAGTCTCGAAG 3′
*MCP-1*	NM_031020.3	**F**	5′ GCTACAAGAGGATCACCAGCAG 3′
		**R**	5′ GTCTGGACCCATTCCTTCTTGG 3′
*18s rRNA*	NR_046237.2	**F**	5′ GTAACCCGTTGAACCCCATT 3′
		**R**	5′ CAAGCTTATGACCCGCACTT 3′

**Table 2:** Docking binding affinity ΔG (kcal/mol).

Receptor	Ligand	Score
BAX-Q63690-F1-model_v6	Cilostazol	−7.4
BAX-Q63690-F1-model_v6	Disulfiram	−3.8
fold_asc_p63116_model_0	Cilostazol	−8.7
fold_asc_p63116_model_0	Disulfiram	−4.2
fold_gsdmd_a0a096mj11_model_0	Cilostazol	−7.4
fold_gsdmd_a0a096mj11_model_0	Disulfiram	−4
fold_kim_1_o54947_model_0	Cilostazol	−6.2
fold_kim_1_o54947_model_0	Disulfiram	−3.5
fold_miRNA_155_model_0	Cilostazol	−8.2
fold_miRNA_155_model_0	Disulfiram	−4.2
JAK2-Q62689-976-model_v6	Cilostazol	−8.2
JAK2-Q62689-976-model_v6	Disulfiram	−4.4
NLRP3-D4A523-520-model_v6	Cilostazol	−9.6
NLRP3-D4A523-520-model_v6	Disulfiram	−4.2

**Table 3:** Interaction table for Cilostazol with docked proteins.

Protein	Interaction	Distance	Category	Type
BAX	A:THR42:N-:CILOSTAZOL1:O	2.9498	Hydrogen Bond	Conventional Hydrogen Bond
BAX	A:THR42:OG1-:CILOSTAZOL1:O	2.89487	Hydrogen Bond	Conventional Hydrogen Bond
BAX	CILOSTAZOL1:H-A:GLN32:O	2.13525	Hydrogen Bond	Conventional Hydrogen Bond
BAX	A:SER60:OG-:CILOSTAZOL1	3.26954	Hydrogen Bond	Pi–Donor Hydrogen Bond
BAX	A:GLN32:CB-:CILOSTAZOL1	3.94111	Hydrophobic	Pi–Sigma
BAX	A:PRO50-:CILOSTAZOL1	4.32191	Hydrophobic	Alkyl
BAX	A:LYS57-:CILOSTAZOL1	3.95559	Hydrophobic	Alkyl
BAX	CILOSTAZOL1-A:PRO50	4.83574	Hydrophobic	Pi–Alkyl
ASC	A:GLY110:N-:CILOSTAZOL1:O	3.09767	Hydrogen Bond	Conventional Hydrogen Bond
ASC	A:SER109:CB-:CILOSTAZOL1:O	3.57429	Hydrogen Bond	Carbon Hydrogen Bond
ASC	A:TYR259-:CILOSTAZOL1	5.25055	Hydrophobic	Pi–Pi T-shaped
ASC	CILOSTAZOL1-A:ARG345	4.54549	Hydrophobic	Pi–Alkyl
gsdmd	A:ARG54:NH2-:CILOSTAZOL1:N	2.90706	Hydrogen Bond	Conventional Hydrogen Bond
gsdmd	A:THR447:OG1-:CILOSTAZOL1:O	3.06147	Hydrogen Bond	Conventional Hydrogen Bond
gsdmd	A:SER448:OG-:CILOSTAZOL1:O	3.2732	Hydrogen Bond	Conventional Hydrogen Bond
gsdmd	A:GLY432:CA-:CILOSTAZOL1:O	3.40946	Hydrogen Bond	Carbon Hydrogen Bond
gsdmd	CILOSTAZOL1:C-A:VAL26:O	3.30702	Hydrogen Bond	Carbon Hydrogen Bond
gsdmd	CILOSTAZOL1:C-:CILOSTAZOL1	3.87877	Hydrophobic	Pi–Sigma
gsdmd	A:ALA6-:CILOSTAZOL1	3.48753	Hydrophobic	Alkyl
gsdmd	A:PRO25-:CILOSTAZOL1	5.44889	Hydrophobic	Alkyl
gsdmd	A:PRO451-:CILOSTAZOL1	4.99197	Hydrophobic	Alkyl
gsdmd	CILOSTAZOL1-A:ALA6	5.30488	Hydrophobic	Pi–Alkyl
gsdmd	CILOSTAZOL1-A:LYS10	3.79093	Hydrophobic	Pi–Alkyl
gsdmd	CILOSTAZOL1-A:PRO25	5.03819	Hydrophobic	Pi–Alkyl
KIM-1	CILOSTAZOL1:H-A:THR122:O	2.41851	Hydrogen Bond	Conventional Hydrogen Bond
KIM-1	A:GLN119:NE2-:CILOSTAZOL1	3.65621	Hydrogen Bond	Pi–Donor Hydrogen Bond
KIM-1	A:PHE123-:CILOSTAZOL1	5.47118	Hydrophobic	Pi–Pi T-shaped
KIM-1	A:MET121-:CILOSTAZOL1	5.31006	Hydrophobic	Alkyl
KIM-1	A:PRO228-:CILOSTAZOL1	4.6014	Hydrophobic	Alkyl
KIM-1	A:ARG230-:CILOSTAZOL1	5.49999	Hydrophobic	Alkyl
KIM-1	CILOSTAZOL1-A:MET121	5.46979	Hydrophobic	Alkyl
KIM-1	CILOSTAZOL1-A:ARG230	4.93199	Hydrophobic	Pi–Alkyl
KIM-1	CILOSTAZOL1-A:MET121	4.71316	Hydrophobic	Pi–Alkyl
miRNA_155	A:A6:N6-:CILOSTAZOL1:N	3.22546	Hydrogen Bond	Conventional Hydrogen Bond
miRNA_155	A:A7:N6-:CILOSTAZOL1:N	2.92348	Hydrogen Bond	Conventional Hydrogen Bond
miRNA_155	A:A7:N6-:CILOSTAZOL1	3.31132	Hydrogen Bond	Pi–Donor Hydrogen Bond
miRNA_155	A:A55:N6-:CILOSTAZOL1	3.8743	Hydrogen Bond	Pi–Donor Hydrogen Bond
JAK2	A:SER936:N-:CILOSTAZOL1:N	3.01422	Hydrogen Bond	Conventional Hydrogen Bond
JAK2	A:GLY993:CA-:CILOSTAZOL1:O	3.67813	Hydrogen Bond	Carbon Hydrogen Bond
JAK2	A:ASP994:OD2-:CILOSTAZOL1	3.86891	Electrostatic	Pi–Anion
JAK2	A:LEU855-:CILOSTAZOL1	4.75935	Hydrophobic	Alkyl
JAK2	A:VAL863-:CILOSTAZOL1	4.82827	Hydrophobic	Alkyl
JAK2	A:ALA880-:CILOSTAZOL1	4.67186	Hydrophobic	Alkyl
JAK2	A:LEU983-:CILOSTAZOL1	4.54139	Hydrophobic	Alkyl
JAK2	CILOSTAZOL1-A:LEU983	5.46039	Hydrophobic	Pi–Alkyl
NLRP3	A:TYR379:OH-:CILOSTAZOL1:N	2.80358	Hydrogen Bond	Conventional Hydrogen Bond
NLRP3	A:ARG152:NH2-:CILOSTAZOL1	4.26714	Electrostatic	Pi–Cation
NLRP3	A:ARG152:NE-:CILOSTAZOL1	3.76105	Hydrogen Bond	Pi–Donor Hydrogen Bond
NLRP3	A:TYR379:OH-:CILOSTAZOL1	3.39309	Hydrogen Bond	Pi–Donor Hydrogen Bond
NLRP3	A:ILE232:CD1-:CILOSTAZOL1	3.69278	Hydrophobic	Pi-Sigma
NLRP3	CILOSTAZOL1-:CILOSTAZOL1	5.1416	Hydrophobic	Pi–Pi T-shaped
NLRP3	A:ARG165-:CILOSTAZOL1	4.80201	Hydrophobic	Alkyl
NLRP3	A:ILE232-:CILOSTAZOL1	4.16394	Hydrophobic	Alkyl
NLRP3	A:PRO410-:CILOSTAZOL1	4.49034	Hydrophobic	Alkyl
NLRP3	CILOSTAZOL1-A:ILE149	4.25367	Hydrophobic	Alkyl
NLRP3	CILOSTAZOL1-A:ILE232	4.97842	Hydrophobic	Alkyl
NLRP3	A:PHE371-:CILOSTAZOL1	5.35995	Hydrophobic	Pi–Alkyl
NLRP3	CILOSTAZOL1-A:ILE232	5.47799	Hydrophobic	Pi–Alkyl
NLRP3	CILOSTAZOL1-A:ARG152	5.26567	Hydrophobic	Pi–Alkyl

## Data Availability

All data are available in this manuscript.
